# Integrated transcriptome and endogenous hormone analysis provides new insights into callus proliferation in *Osmanthus fragrans*

**DOI:** 10.1038/s41598-022-11801-9

**Published:** 2022-05-09

**Authors:** Heng Gu, Wenjie Ding, Tingting Shi, Qixia Ouyang, Xiulian Yang, Yuanzheng Yue, Lianggui Wang

**Affiliations:** 1 Key Laboratory of Landscape Architecture, Nanjing Forestry University, Nanjing, Jiangsu China; 2 College of Landscape Architecture, Nanjing Forestry University, Nanjing, China; 3 Co-Innovation Center for Sustainable Forestry in Southern China, Nanjing Forestry University, Nanjing, China

**Keywords:** Molecular biology, Plant sciences

## Abstract

*Osmanthus fragrans* is an important evergreen species with both medicinal and ornamental value in China. Given the low efficiency of callus proliferation and the difficulty of adventitious bud differentiation, tissue culture and regeneration systems have not been successfully established for this species. To understand the mechanism of callus proliferation, transcriptome sequencing and endogenous hormone content determination were performed from the initial growth stages to the early stages of senescence on *O*. *fragrans* calli. In total, 47,340 genes were identified by transcriptome sequencing, including 1798 previously unidentified genes specifically involved in callus development. Kyoto Encyclopedia of Genes and Genomes (KEGG) pathway analysis of differentially expressed genes (DEGs) was significantly enriched in plant hormone signal transduction pathways. Furthermore, our results from the orthogonal projections to latent structures discrimination analysis (OPLS-DA) of six typical hormones in five development stages of *O*. *fragrans* calli showed jasmonic acid (JA) could play important role in the initial stages of calli growth, whereas JA and auxin (IAA) were dominant in the early stages of calli senescence. Based on the weighted gene co-expression network analysis, *OfSRC2*, *OfPP2CD5* and *OfARR1*, *OfPYL3*, *OfEIL3b* were selected as hub genes from the modules with the significant relevance to JA and IAA respectively. The gene regulation network and quantitative real-time PCR implied that during the initial stages of callus growth, the transcription factors (TFs) *OfERF4* and *OfMYC2a* could down-regulate the expression of hub genes *OfSRC2* and *OfPP2CD5*, resulting in decreased JA content and rapid callus growth; during the late stage of callus growth, the TFs *OfERF4*, *OfMYC2a* and *OfTGA21c*, *OfHSFA1* could positively regulate the expression of hub genes *OfSRC2*, *OfPP2CD5* and *OfARR1*, *OfPYL3*, *OfEIL3b*, respectively, leading to increased JA and IAA contents and inducing the senescence of *O*. *fragrans* calli. Hopefully, our results could provide new insights into the molecular mechanism of the proliferation of *O*. *fragrans* calli.

## Introduction

*Osmanthus fragrans* is one of the top-ten traditional Chinese flowers, with both ornamental and medicinal value, which is widely distributed in southern China and the other subtropical regions of the world^1,2^. Through a long evolution, *O*. *fragrans* has been divided into five groups^3^. In our previous research, the genome of *O*. *fragrans* was sequenced using third-generation sequencing technology^4^. Publication of the whole genome of *O*. *fragrans* enabled researchers to speed up the identification of key ornamental genes in this species^5^. However, the function of those key genes remains to be studied because of the lack of a genetic transformation system. Basic research into woody plants has lagged behind that of herbaceous plants because of their inherent biological characteristics^6,7^. Previous studies have shown that adventitious buds are difficult to differentiate from calli in many plants, such as *Cyclocarya paliurus*, *Paeonia suffruticosa*, and *Paeonia lactiflora*^8,9^. An efficient and stable regeneration system of plant tissue culture is the basis of genetic transformation, and thus, it will be of significance to establish such a system for *O*. *fragrans*.

In vitro culture of *O*. *fragrans* callus is mainly induced by explants of somatic embryo, leaves, and stem tips^10^, with calli showing low proliferation efficiency in such cultures. Callus proliferation could be improved by optimizing hormone concentrations and culture conditions. The efficiency of callus proliferation directly affects the in vitro regeneration system, which is essential for the establishment of a genetic transformation system, therefore, callus proliferation is an important process in the genetic transformation system of *O*. *fragrans*. However, calli show slow growth, low proliferation efficiency, and difficulties in dedifferentiating in tissue cultures of *O*. *fragrans*, which might be related to their endogenous hormone content. Plant hormones have an important role in the regulation of cell proliferation, division, structure, differentiation, and metabolism. Auxin (IAA) can promote cell elongation and maintain apical dominance, which plays an important role in plant growth and development^11^. Gibberellin (GA_3_) can promote cell growth and leaf root development. When plants are in stress, abscisic acid (ABA) content will increase, which enables plants to grow normally^12^. During the growth and development of plant tissues, jasmonic acid (JA) can promote the activation and regeneration of stem cells, while brassinosteroids (BR) can promote the growth of buds^13,14^. In addition, zeatin (ZR), as a component of cytokinin, plays a physiological role in promoting cell division and preventing senescence^15^. Callus dedifferentiation is closely related to the types and concentrations of endogenous hormone in plants^16^. Adventitious buds can only be differentiated from plant callus under proper hormone concentrations. Therefore, there is a need to identify the key endogenous hormones and regulator pathways involved in the growth of *O*. *fragrans* calli.

Recently developed bioinformatics and large-scale sequencing platforms have reduced sequencing costs and cycle times, and are widely used in transcriptome studies^17^. Transcriptome sequencing technologies provide a range of information, including gene domain functional annotation, variable cleavage, transcriptional sequencing, differential gene expression analysis, and gene function classification^18^. It is possible to quickly and efficiently analyze differentially expressed genes and functionally annotate regulatory pathways by using transcriptome sequencing. This approach has been widely used in the study of plant calli. For example, in *Citrus reticulata*, key genes inducing carotenoid degradation were identified through transcriptome sequencing of calli^19^. After treating fresh petioles with a browning antagonist, transcriptomic sequencing was performed on the calli of peony petioles, and key genes related to browning were identified^20^. In addition, in a transcriptome sequencing study of melons, important genes that promote the differentiation of melons from nonembryogenic calli into embryogenic calli were also identified^18^. However, there are no reports on transcriptional aspects of *O*. *fragrans* calli.

The proliferation and differentiation of calli are complex processes, regulated by gene expression^21,22^. In some model plants, the molecular mechanisms of callus proliferation and differentiation have been revealed. In *Arabidopsis thaliana*, *LBD16* is specifically expressed in newly formed callus and the *WOX11*-*LBD16* pathway can promote callus cells to acquire totipotency^23^. Auxin can stabilize the expression of *AtbZIP59* and, if *AtbZIP59* is interrupted, it can inhibit the formation of auxin-induced calli, whereas the overexpression of *AtbZIP59* can promote callus formation^24^. Overexpression of *PtWOX11* not only regulated the regeneration of new shoots, but also promoted the formation of calli, providing a means of genetic engineering for poplar reproduction^25^. In addition, a recent study found that *MYB94* and *MYB96* directly inhibit *AtLBD29* expression, with plants overexpressing this gene showing obvious phenotypic changes during vegetative growth, which in turn inhibited callus formation^26^. However, genes related to callus proliferation and differentiation in *O*. *fragrans* have not been reported, and the molecular mechanisms involved remain unclear.

To address the low proliferation efficiency of callus and difficulty in callus dedifferentiation in *O*. *fragrans*, we determined the endogenous hormone content of *O*. *fragrans* calli at different growth stages and then performed transcriptome sequencing. Weighted gene co-expression network analysis (WGCNA) of endogenous hormones and the transcriptome was performed to identify key genes involved in the regulating of *O*. *fragrans* callus proliferation. This study lays the foundation for elucidating the molecular mechanism of callus proliferation in *O*. *fragrans*.

## Results

### Endogenous hormone content of *O. fragrans* callus

**Figure 1:**
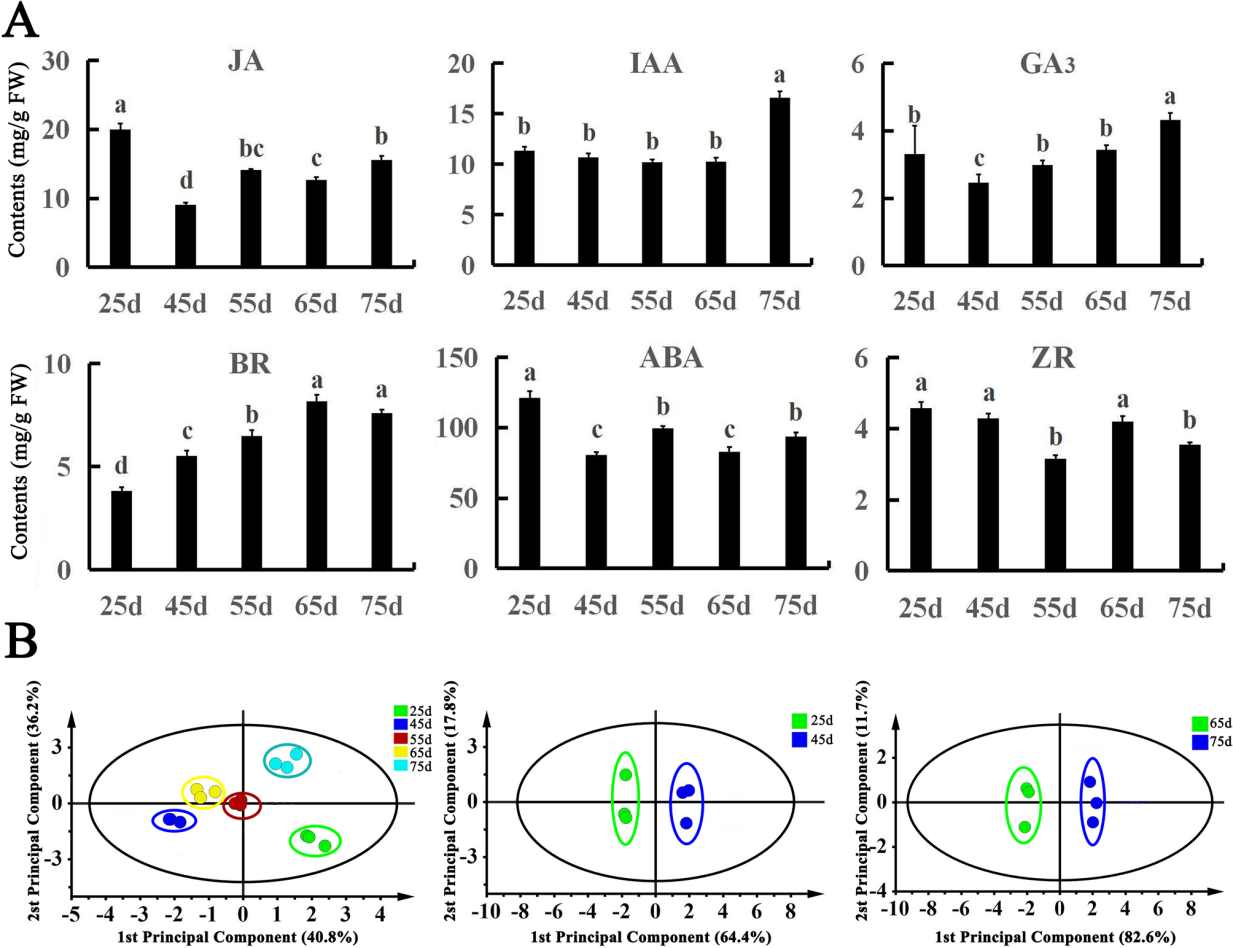
Analysis of endogenous hormone content in callus of *O. fragrans* at different developmental stages. (**A**) Content of six endogenous hormones in callus of *O. fragrans* at five developmental stages. *ABA* abscisic acid, *BR* brassinosteroids, *GA*_*3*_ gibberellin, *IAA* auxin, *JA* jasmonic acid, *ZR* zeatin. Different letters denote significant differences according to the Tukey’s test (*P* < 0.05). (**B**) Score plot of endogenous hormone content of *O*. *fragrans* according to different developmental stages using OPLS-DA analysis.

Levels of endogenic hormones, including JA, IAA, GA_3_, ABA, BR, and ZR, were determined in the callus of *O*. *fragrans* at different development stages by enzyme linked immunosorbent assay (ELISA) (Fig. 1A). With increases in growing days, the GA_3_ content in the callus of *O*. *fragrans* first decreased and then increased, with the lowest content at 45 days and the highest at 75 days. The IAA content in calli at 25 days, 45 days, 55 days, and 65 days was unchanged, although the content suddenly increased at 75 days, which was significantly higher than in the other stages. The BR content peaked at 65 days of growth, and then decreased, being significantly different from that at 25 days, 45 days, and 55 days, but not at 75 days. The ABA content in calli was high at all five developmental stages examined, peaking at 121.12 ng/g·FW at 25 days, which was significantly different from that at other stages. The expression of JA and ZR first decreased, then increased, and then decreased again. The JA content had increased by 55 days, decreased at 65 days and increased again at 75 days, whereas the ZR content was decreased at 55 days, increased at 65 days, and decreased at 75 days.

Simca14.1 software was used for Orthogonal projections to latent structures discrimination analysis (OPLS-DA) of the six endogenous hormones measured in the callus of *O*. *fragrans*. Data points for the six hormones fell within the 95% confidence interval (CI), and the biological repeated data points in the same period had relatively clear clustering, with data points associated with the different developmental stages clustering in different regions (Fig. 1B). According to the highest value of variable important in projection (VIP) , JA was the main endogenous hormone during the initial stages of callus growth, whereas JA and IAA were the major endogenous hormones during the early stages of callus senescence (Table S1).

### Transcriptome sequencing results

**Figure 2:**
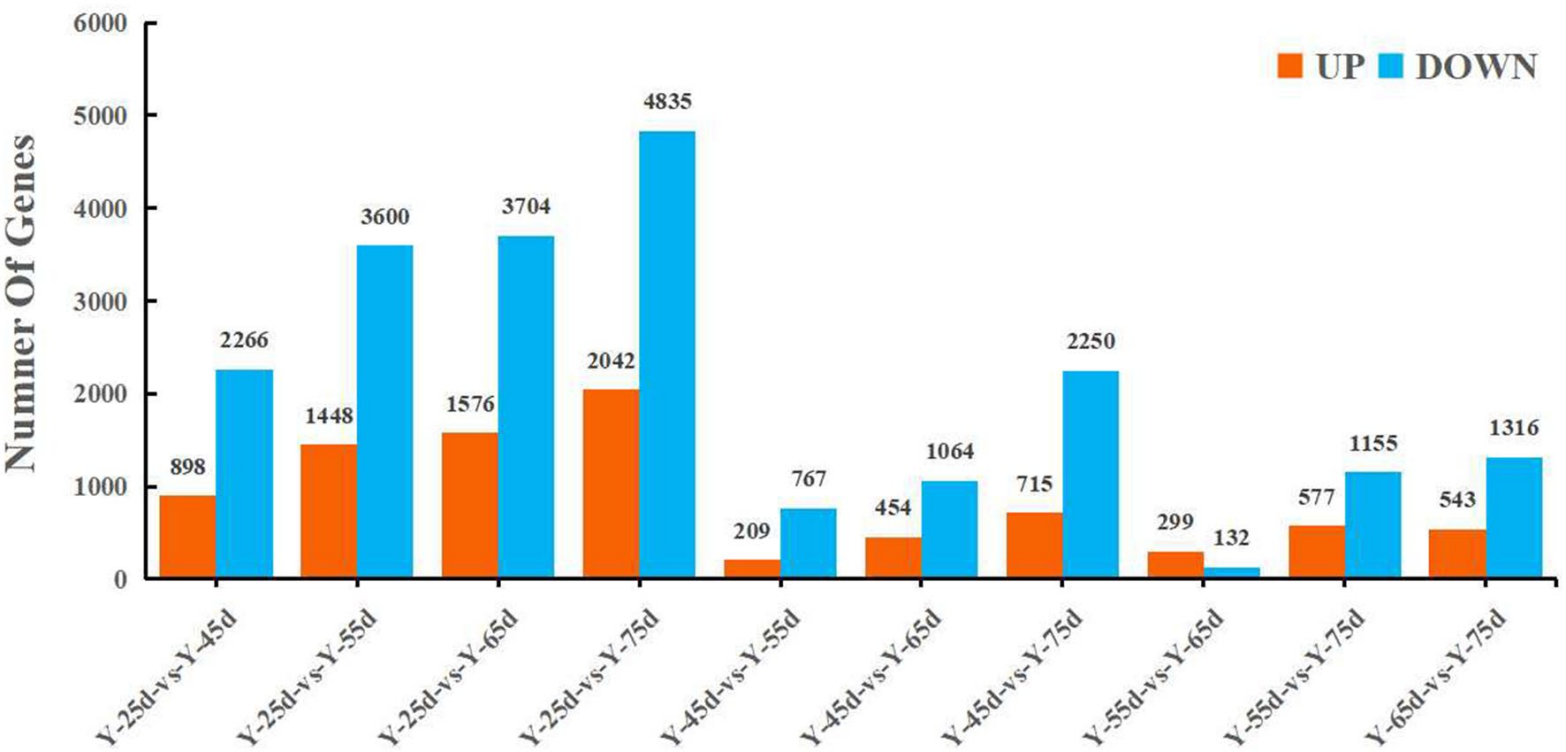
Number of DEGs. Y-25d: callus at 25 days; Y-45d: callus at 45 days; Y-55d: callus at 55 days; Y-65d: callus at 65 days; Y-75d: callus at 75 days.

In total, 15 samples of calli from *O*. *fragrans* at different growth stages were sequenced, resulting in 622,892,416 raw reads and 621,588,326 clean reads (Table 1). The GC content of each sample was > 45%. The percentage of Q20 was 96% or more, whereas that of Q30 was 91% or more. In this study, 1798 previously unidentified genes were obtained by comparing our data with the whole genome of *O*. *fragrans*. Differentially expressed genes (DEGs) during the callus growth were compared, revealing 3164 significantly different genes between 25 days versus 45 days, including 898 upregulated genes and 2266 downregulated genes (Fig. 2). However, there were 1895 significantly differently expressed genes between 65 days versus 75 days, of which 543 were upregulated and 1316 were downregulated (Fig. 2).

**Table 1:** Summary of sequencing data.

Sample	Raw reads	Clean reads	GC (%)	Q20 (%)	Q30 (%)
Y-25d-1	38,273,044	38,202,528	45.21	97.19	92.24
Y-25d-2	47,606,498	47,523,558	45.53	97.23	92.33
Y-25d-3	40,497,308	40,423,992	45.35	97.18	92.24
Y-45d-1	36,949,338	36,870,174	45.23	97.10	92.11
Y-45d-2	45,167,060	45,072,312	45.19	97.14	92.16
Y-45d-3	41,770,322	41,682,140	45.36	97.33	92.64
Y-55d-1	46,146,066	46,046,912	45.31	97.00	91.85
Y-55d-2	36,084,422	36,003,466	45.59	97.16	92.27
Y-55d-3	40,747,532	40,649,052	45.27	96.87	91.64
Y-65d-1	44,495,046	44,403,632	45.35	96.95	91.73
Y-65d-2	44,108,678	44,020,824	45.06	97.31	92.56
Y-65d-3	42,253,820	42,163,112	45.32	97.15	92.16
Y-75d-1	36,028,678	35,952,558	45.51	97.50	93.03
Y-75d-2	39,943,210	39,846,950	45.23	97.08	92.07
Y-75d-3	42,821,394	42,727,116	45.06	97.07	92.06

*Y-25d* callus at 25 days, *Y-45d* callus at 45 days, *Y-55d* callus at 55 days, *Y-65d* at 65 days, *Y-75d* callus at 75 days, *Raw reads* original number of reads obtained by sequencing, *Clean reads* number of reads after removing low-quality reads and trimming adapter sequences, *GC%* percentage of G and C in total bases, *Q20* Phred score, indicates 99% accuracy of sequenced bases, *Q30* Phred score, indicates 99.9% accuracy of sequenced bases.

### Identification and functional annotation of DEGs in callus proliferation of *O. fragrans*

Gene Ontology (GO) classifications and Kyoto Encyclopedia of Genes and Genomes (KEGG) pathway analyses of DEGs in calli of *O*. *fragrans* were carried out to compare of five development stages. Four growth periods with significant differences between endogenous hormones were selected for comparison: 25 days versus 45 days and 65 days versus 75 days. The GO classification of DEGs in *O*. *fragrans* callus mainly focused on molecular functions, cellular components, and biological processes. DEGs were enriched in 45 GO terms at 25 days versus 45 days (Fig. S1). Biological processes were significantly enriched in 21 pathways, including metabolic processes, biological attachment, and cell death. Cell components were significantly enriched in 14 pathways, such as membrane, cell, and whole-membrane envelopment. Molecular functions were significantly enriched in ten pathways, including signal transduction activity, transport activity, and molecular regulation activity. DEGs were enriched on 46 GO terms at 65 days versus 75 days (Fig. S2). Biological processes were significantly enriched in 21 ways, such as metabolic processes, cellular processes, and single cell processes. Cell components were significantly enriched in 15 pathways, such as cell component organelles. Molecular functions were significantly enriched in ten pathways, such as catalytic activity combined with transport activity.

There were significant differences in the KEGG pathways of DEGs in calli of *O*. *fragrans*. At 25 days versus 45 days, 631 genes were enriched in 111 metabolic pathways and were significantly enriched in 17 metabolic pathways, involving mainly plant hormone signaling transduction, photosynthesis-antennae proteins, and biosynthesis of phenylpropanol (Fig. S3). At 65 days versus 75 days, 396 genes were enriched in 100 metabolic pathways, with significant enrichment in eight metabolic pathways, including plant hormone signaling transduction, phenylpropanol biosynthesis, and plant circadian rhythm (Fig. S4).

### Identification of DEG co-expression modules by WGCNA

**Figure 3:**
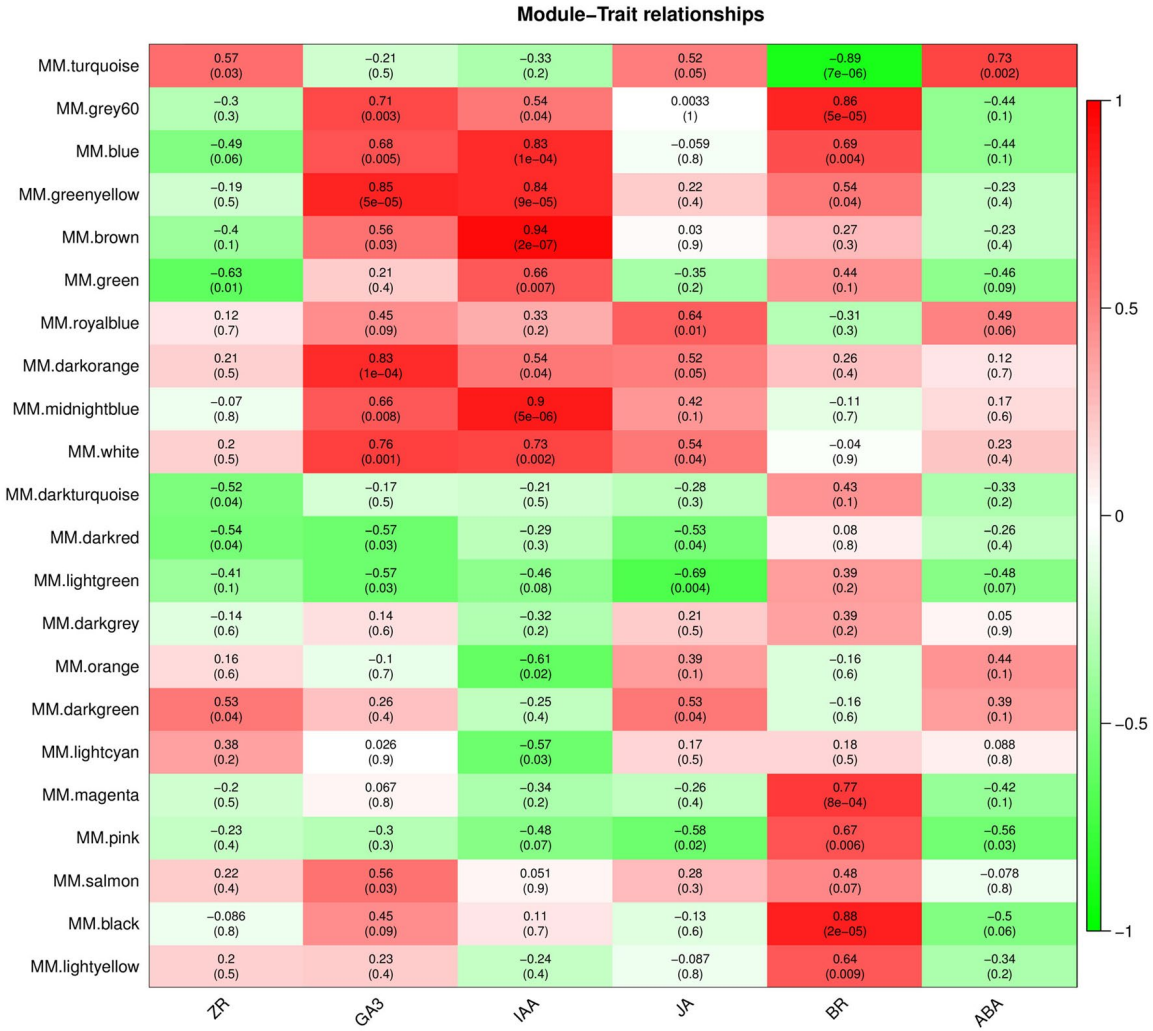
Thermal diagram of endogenous hormones and characteristic modules. Each column represents a physiological indicator, and each row represents a genetic module. The number in each grid represents the correlation between the module and the trait. The number in parentheses represents the *P*-value. The smaller the *P*-value, the stronger the significance of the representativeness and module correlation. *ABA* abscisic acid, *BR* brassinosteroids, *GA*_*3*_ gibberellin, *IAA* auxin, *JA* jasmonic acid, *ZR* zeatin.

RNA-seq sequencing was used to analyze the DEGs of callus proliferation. To further identify the hub genes that regulate the proliferation of *O*. *fragrans* calli, WGCNA was also performed on the endogenous content hormones and the transcriptome (Fig. 3). The IDs of selected genes were shown in Table S2. By screening the weight values, a total of 22 co-expression modules was obtained, each containing a set number of genes. First, the upregulated characteristic module ‘royalblue’ corresponding to the endogenous hormone JA was used to highlight genes in the signal transduction pathway of plant hormones. Then, according to the Module Membership (MM) value, hub genes were screened and included *OfSRC2* and *OfPP2CD5*. Finally, from the upregulated module ‘brown’, according to the MM value, six hub genes related to IAA were screened out: *OfPP2CA*, *OfPYL3*, *OfEIL3a*, *OfEIL3b*, *OfAHK3*, and *OfARR1*.

### Verification of gene expression through quantitative real-time PCR (qRT-PCR)

**Figure 4:**
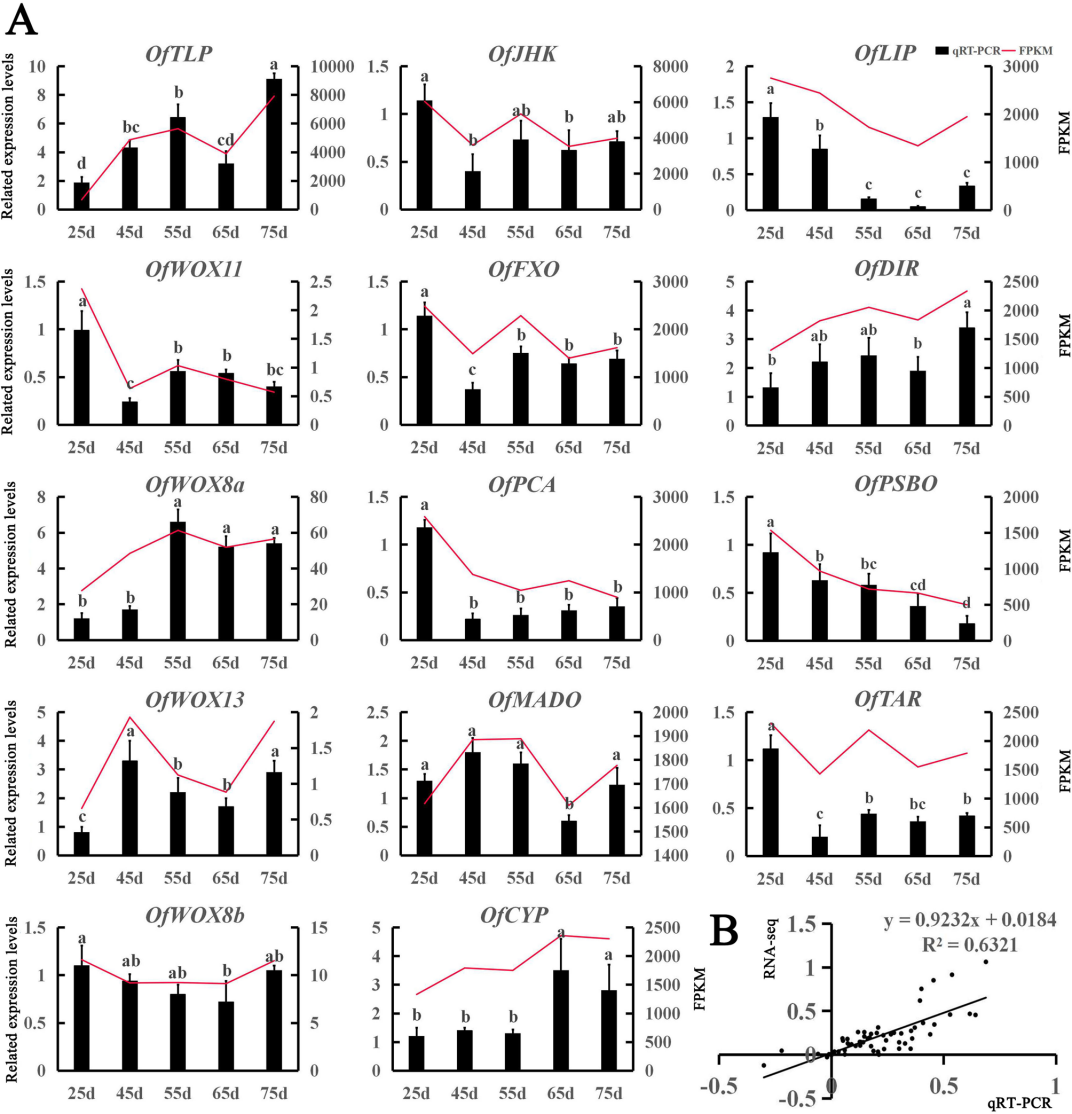
qRT-PCR validation of the transcriptome data results for 14 selected genes. (**A**) Expression levels of 14 genes and FPKM values. Different letters denote significant differences according to the Tukey’s test (*P* < 0.05). (**B**) Correlation analysis of the gene expression ratios between qRT-PCR and FPKM.

To verify the results of transcriptome sequencing, 14 genes were randomly selected from the transcriptome results for qRT-PCR validation. The trends in qRT-PCR expression were consistent with those of the FPKM values (Fig. 4A). However, some genes showed different trends, possibly because of differences between expression detection in qRT-PCR versus transcriptome sequencing. Further correlation analysis showed that a correlation coefficient of R^2^ = 0.6321, indicating that the transcriptome data were accurate and reliable, and could be used in subsequent experiments (Fig. 4B).

### The co-expression network reveals the regulatory network of endogenous hormones JA and IAA

**Figure 5:**
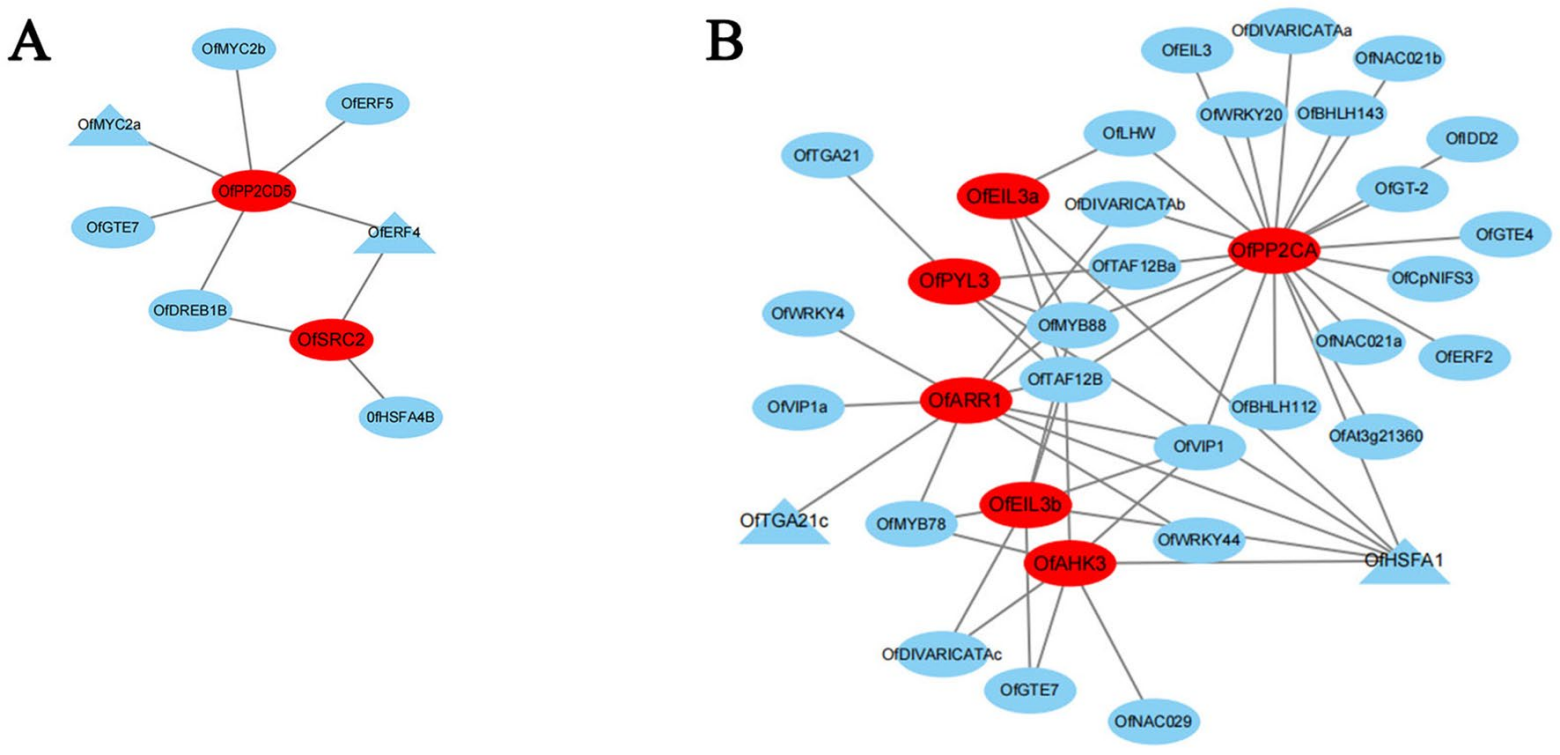
Regulatory networks of hub genes and edge genes in feature modules. (**A**) Construction of a regulatory network of JA-related upregulated genes and transcription factors in ‘royalblue’ module. (**B**) Construction of a regulatory network of IAA-related upregulated genes and transcription factors in the ‘brown’ module. Hub genes are red ellipses, transcription factors are blue ellipses, and TFs with the highest correlation are blue triangles.

**Figure 6:**
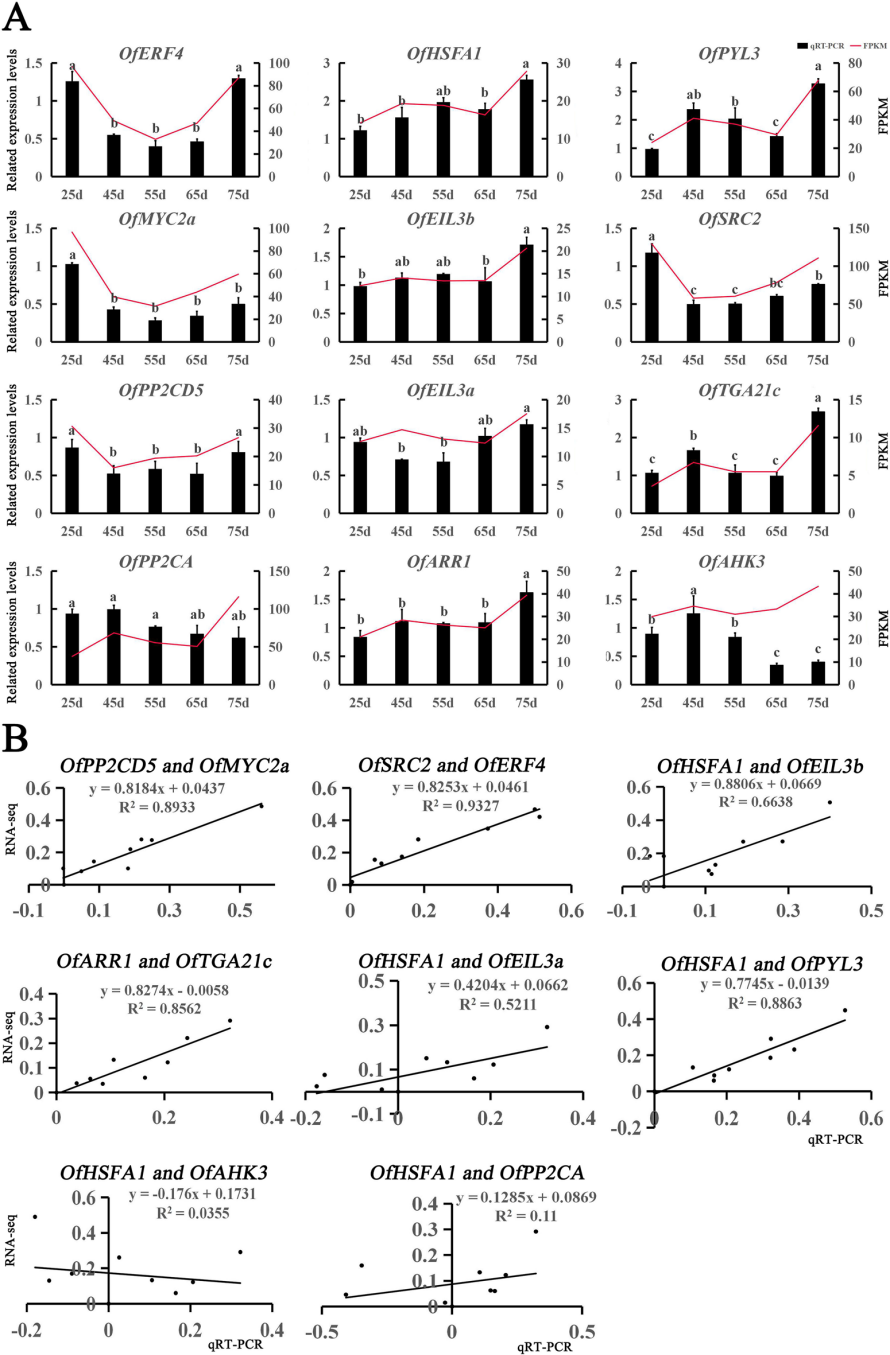
qRT-PCR validation of hub genes and edge genes in feature modules. (**A**) The expression levels of 12 genes (four genes from the ‘royalblue’ module and eight from the ‘brown’ module) and FPKM values. Different letters denote significant differences according to the Tukey’s test (*P* < 0.05). (**B**) Correlation coefficient analysis of hub genes and edge genes in feature modules.

To understand the regulatory network relationship between hub genes and transcription factors (TFs) in JA and IAA, TFs corresponding to two key genes in ‘royalblue’, the upregulated module corresponding to JA, were screened. In addition, in ‘brown’, the upregulated module corresponding to IAA, TFs corresponding to six hub genes were screened, and network regulation maps were drawn. Visualized in Cytoscape, nine nodes in the endogenous hormone JA regulatory network were connected to nine edges (Fig. 5A), and 35 nodes in the IAA regulatory network were connected to 55 edges (Fig. 5B). Then, 12 genes, including hub genes and corresponding TFs, were analyzed by qRT-PCR (Fig. 6A) using the primers listed in Table S3. Using R^2^ > 0.6 as the standard, five pairs of combinations were screened out with interaction relationships: *OfMYC2a* and *OfPP2CD5*, *OfERF4* and *OfSRC2*, *OfHSFA1* and *OfEIL3b*, *OfTGA21c* and *OfARR1*, and *OfHSFA1* and *OfPYL3* (Fig. 6B).

**Figure 7:**
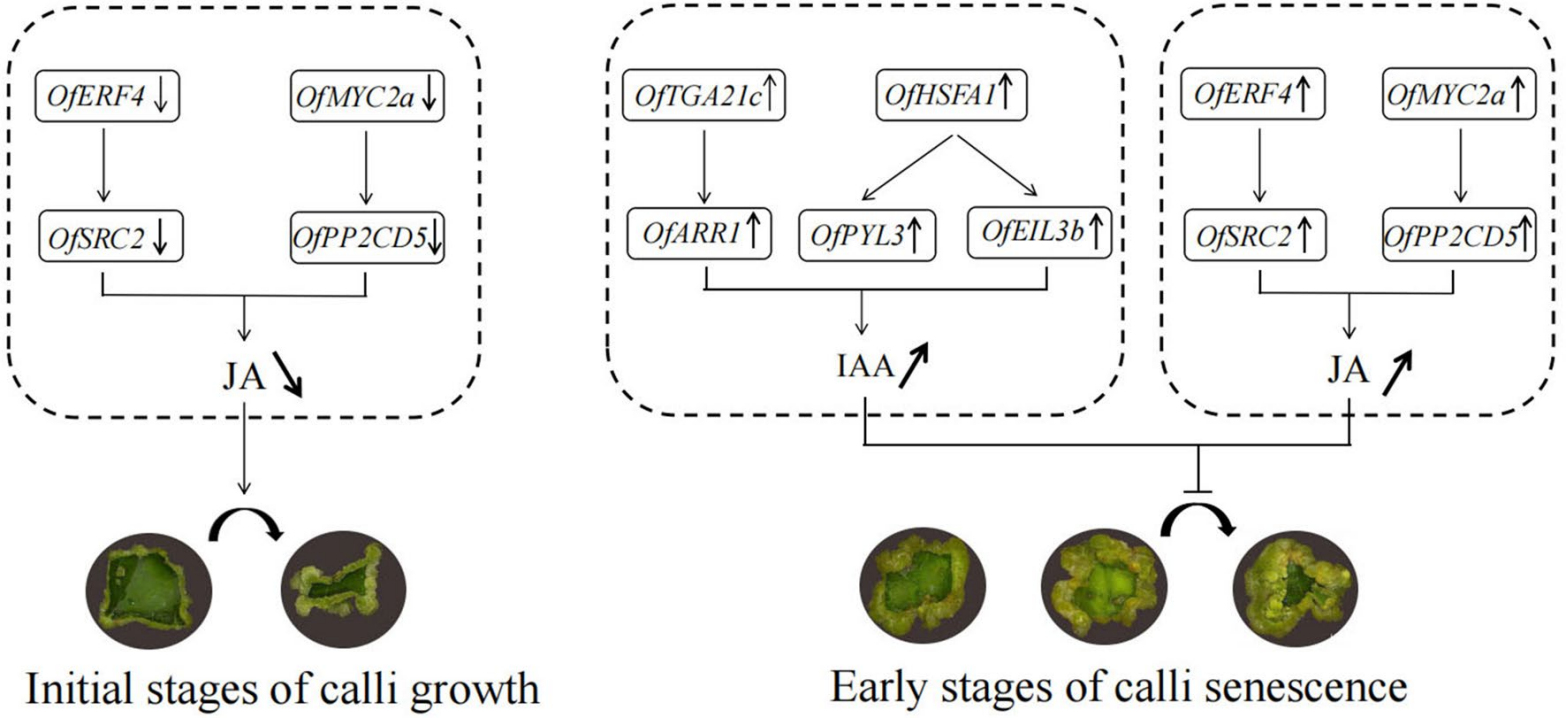
Proposed model of callus proliferation regulation in *O. fragrans*.

Given these results, we propose a model based on changes in hormone content and network regulation maps during callus proliferation in *O. fragrans* (Fig. 7). During the initial growth stages, TFs (*OfERF4* and *OfMYC2a*) could negatively regulate the hub genes (*OfSRC2* and *OfPP2CD5*), decreasing the JA content and leading to rapid callus growth. During the early stages of calli senescence, the TFs *OfTGA21c* and *OfHSFA1* could positively regulate hub genes (*OfARR1* and *OfPYL3*, *OfEIL3b*) and the hub genes (*OfSRC2* and *OfPP2CD5*) could also regulated by TFs *OfERF4* and *OfMYC2a* respectively, leading to increased IAA and JA contents and the senescence of *O*. *fragrans* calli.

## Discussion

**Figure 8:**
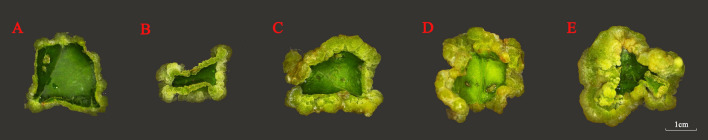
Different developmental stages of *O. fragrans* callus. Callus at (**A**) 25 days, (**B**) 45 days, (**C**) 55 days, (**D**) 65 days, and (**E**) 75 days of growth.

The amount and type of endogenous hormones have an important influence on the growth and development of plants^27^, such as seed germination^28^, cell differentiation^29^, root growth^30^, and fruit ripening and shedding^31^. In the tissue culture of *O. fragrans* calli, we find that the calli experienced from the initial growth stages to the early stages of calli senescence. JA and IAA are important hormones involved in the regulation of many physiological processes of plant growth and development^32–34^. The five growth stages of callus formation were divided according to the formation time and development status of *O. fragrans* callus (Fig. 8). Each growth stage was regulated by a variety of endogenous hormones. The content of endogenous hormones in calli of *O*. *fragrans* during these stages was determined. During the growth period from 25 to 45 days, the JA content decreased gradually (Fig. 1A), and the calli grew rapidly at this time, indicating that a low concentration of JA promotes the rapid proliferation of *O*. *fragrans* calli, similar to results of studies on callus proliferation in *Allium sativum*^35^. In general, IAA can promote the growth of plant callus. In this study, the content of IAA remained unchanged from 25 to 65 days (Fig. 1A), but increased suddenly from 65 to 75 days, suggesting that the increased IAA concentration inhibited callus growth. The possible reason was that a certain level of IAA could promote the proliferation of callus, while higher levels of IAA could exert an inhibitory effect on this process, which was consistent with the results of previous studies^36^. These results suggested that the changes of JA and IAA contents affected the calli growth of *O*. *fragrans*.

Endogenous hormones can coregulate the growth and development of plant organs^37,38^. The current study showed that endogenous hormones have a leading role from the initial growth stages to the early stages of calli senescence (Fig. 1B). The results from OPLS-DA and the highest value of VIP showed JA to be the dominant endogenous hormone during the initial stages of calli growth, whereas JA and IAA were dominant during the early stages of calli senescence (Fig. 1B and Table S1). Interestingly, IAA and JA showed similar changes in concentration during the early stages of calli senescence (Fig. 1A), suggesting that they have synergistic effects and jointly inhibit callus growth. The callus with high endogenous IAA content had a higher emergence rate, whereas decreases in IAA content promoted callus dedifferentiation. The content of IAA at 75 d was significantly higher than that at other stages (Fig. 1A), suggesting that the IAA content should be reduced during the later stages of the culture process to enable callus dedifferentiation.

Transcriptome sequencing was performed on different development stages of callus of *O*. *fragrans*. In total, 47,340 genes were obtained by transcriptome sequencing, and 1798 previously unidentified genes were found by comparison with the whole *O*. *fragrans* genome. These latter genes might be related to the proliferation and differentiation of *O*. *fragrans* calli. The KEGG pathway analysis of DEGs showed that these genes were significantly enriched in pathways, including plant hormone signal transduction (Figs. S3, S4). The transcriptome sequencing of maize embryogenic calli showed that DEGs were mainly related to photosynthesis and plant hormone signal transduction^39^. Similarly, 1418 DEGs were identified by transcriptome sequencing from embryogenic and nonembryogenic calli of *Picea spruce*, mainly involved in plant hormone signal transduction and other pathways^40^. From plant hormone signal transduction pathways, many genes, including some candidate genes that regulate callus proliferation in *O*. *fragrans*, were identified. These results provide an interesting direction for the future study of *O*. *fragrans* calli.

WGCNA describes the relationship between genes and samples, which can further identify key genes in signaling pathways. Hub genes related to anther development in cotton were identified by WGCNA^41^. Similarly, WGCNA on endogenous hormones and the transcriptome of *O*. *fragrans* callus identified modules corresponding to the upregulation of JA and IAA (Fig. 3), revealing five hub genes in two characteristic modules: two hub genes (*OfSRC2* and *OfPP2CD5*) in the JA pathway and three hub genes (*OfARR1*, *OfPYL3*, and *OfEIL3b*) in the IAA pathway (Fig. 5A,B). Endogenous hormones form a complex signaling network in plants, influencing the growth and development of cells by regulating key genes involved in cell proliferation through endogenous signal transmission^42–44^. Plants can respond quickly when exposed to external stimuli, resulting in changes in the transcription level of numerous genes. Therefore, a large number of TFs are involved in responding to external stress. Furthermore, we identified that the expression trend of hub genes and TFs by qRT-PCR (Fig. 6A). Finally, we screened out five pairs of combinations with interaction relationships: *OfMYC2a* and *OfPP2CD5*, *OfERF4* and *OfSRC2*, *OfHSFA1* and *OfEIL3b*, *OfTGA21c* and *OfARR1*, and *OfHSFA1* and *OfPYL3* (Fig. 6B). These results imply that hub genes are mainly regulated by TFs, changing the content of JA and IAA, and regulating the proliferation of *O*. *fragrans* calli.

In this study, a total of four candidate TFs (*OfMYC2a*, *OfERF4*, *OfTGA21c*, and *OfHSFA1*) which related to the calli formation were identified and used for the qRT-PCR analysis. However, the homologous genes of the reported calli formation related TFs (bZIP, WOX, and MYB) were not found in the feature modules, which indicated that the gene regulatory mechanism of calli formation in *O. fragrans* could be different with the model species. MYC TFs are activators or suppressors of JA gene expression, which regulates plant growth and development^45^. Cloning of the *AtMYC2* homologous gene *MdMYC2* showed that *MdMYC2* was involved in the regulation of JA signaling, resulting in the production of more anthocyanins in overexpressed apple calli^46^. ERF TFs occur in plants and are involved in plant responses to biological and abiotic stresses^47^. In *Taxus*, taxol biosynthesis is regulated by ERF TFs dependent on JA signal transduction^48^. In the current study, early callus growth was rapid (Fig. 7), probably because of TFs *OfMYC2a* and *OfERF4* acting as suppressor factors in response to gene expression, downregulating the expression of hub genes (*OfPP2CD5* and *OfSRC2*) and resulting in decreased JA content and rapid callus growth. TGA TFs are members of the bZIP family and have an important role in the development of stress tolerance^49^. In *A. thaliana*, TGA TFs enhance resistance by regulating the auxin signaling pathway^50^. HSFA is a heat shock TF involved in regulating the expression of cold, heat, high salt, and other stress-related genes to improve plant stress tolerance^51^. In rice, *OsHSFA3* is not only involved in the regulation of plant heat resistance, but also improves plant resistance by regulating the biosynthesis of abscisic acid and reactive oxygen species^52^. The current study also showed that the callus grew slowly or even stopped growing during the early stages of calli senescence (Fig. 7), probably because TFs *OfERF4* and *OfMYC2a* activated the expression of hub genes (*OfSRC2* and *OfPP2CD5*), leading to increases in JA content. At the same time, *OfTGA21c* and *OfHSFA1* also activated the expression of hub genes (*OfARR1* and *OfPYL3*, *OfEIL3b)*, leading to increases in IAA content, inhibiting the growth of *O*. *fragrans* calli.

## Materials and methods

### Plant materials

The plant material for this experiment was *O*. *fragrans* ‘Rixiang Gui’ and the collection of the plant material complied with relevant institutional, national and international guidelines and legislation. Young leaves (the first pair of leaves) of cutting seedlings of *O*. *fragrans* ‘Rixiang Gui’, which were 2 years old and cultivated in greenhouses at Nanjing Forestry University, were first washed with dishwashing liquid to remove attachments on the surface. They were then washed with pure water in a beaker. The leaves were subjected to dark treatment at 4 °C for 2 h and washed with running water for 1 h. Then, the leaves were transferred to an ultra-clean workbench for disinfection. The explants were subjected to 75% ethanol treatment for 30 s and were rinsed in sterile water three times. Subsequently, they were placed in 5% NaClO for 6 min and then rinsed with sterile water five to six times. The leaf edges and veins were then removed with a sterile scalpel, and the remaining leaves were cut into 0.5 × 0.5 cm pieces and inoculated in induction medium comprising MS (Murashige and Skoog medium, Duchefa, The Netherlands) + 6-BA (6-benzylaminopurine, Biosharp, China) 2.0 mg/L + NAA (naphthaleneacetic acid, Biosharp, China) 0.1 mg/L. The medium was changed every 30 days.

Calli of *O*. *fragrans* ‘Rixiang Gui’ at 25 days, 45 days (20 d after the callus began to appear), 55 days (30 days after the callus began to appear), 65 days (40 days after the callus began to appear), and 75 days (50 days after the callus began to appear) from the initial growth stages to the early stages of senescence were removed (Fig. 8) and cut with a sterile scalpel on an ultra-clean workbench. Each callus was transferred to a sterile enzyme-free centrifuge tube with sterile forceps, and immediately frozen in liquid nitrogen for 5 min. All samples were stored at −80 °C. Three samples were collected for each growth period.

### Endogenous hormone content measurements

A total of 15 samples across the five growth stages were sent to China Agricultural University for measurement of endogenous hormone contents. The callus (0.5 g) of each treatment were well-powdered using liquid nitrogen and then samples were crushed by cold methanol. The extract was achieved using 2 mL of 80% methanol at 4 °C for 4 h. Then, the samples were centrifuged for 10 min at 6000 rpm/min to absorb the supernatant. The supernatant was extracted on a C-18 SPE column and evaporated to dryness in a rotary evaporator. After that, the samples were resuspended in 1 ml of sample diluent [500 mL PBS (phosphate buffered saline, composed of 0.1 M phosphate buffer containing 0.9% NaCl, pH 7.5), 0.5 mL Tween-20, 0.5 g gelatin]. Plant hormones were determined by ELISA^53^. Coating antigens and monoclonal antibodies of endogenous hormone (JA, IAA, GA_3_, ABA, BR, and ZR) used for the determination were provided by China Agricultural University. Standards were obtained from Sigma (St Louis, MO, USA). Three biological replicates were used for each sample. SPSS software (version 22.0) was used for data analysis. SIMCA 14.1 software was used to conduct OPLS-DA on six endogenous hormones in the callus of *O*. *fragrans* and to further determine the primary endogenous hormones with a role in different development stages of the calli.

### RNA extraction, cDNA library preparation, and sequencing

Total RNA was extracted from the 15 samples using an RNA Purification Kit (Invitrogen, Carlsbad, CA, USA). The integrity of the RNA was verified by RNase-free agarose gel electrophoresis and the concentration was measured using a Nano Drop 2000 spectrophotometer (Thermo Fisher Scientific, Waltham, MA, USA). The enriched mRNA was then fragmented into short fragments using fragmentation buffer and reverse transcribed into cDNA with random primers. Second-strand cDNAs were synthesized by DNA polymerase I, RNase H, dNTP, and buffer. The the cDNA fragments were then purified with a QiaQuick PCR extraction kit (Qiagen, Venlo, The Netherlands) and end repaired, poly(A) was added, and the fragments were ligated to Illumina sequencing adapters. The ligation reaction was purified with the AMPure XP Beads (1.0X). Ligated fragments were subjected to size selection by agarose gel electrophoresis and amplified by polymerase chain reaction (PCR). For each stage of *O*. *fragrans* callus development, three RNA samples were used to construct a cDNA library and for Illumina Novaseq6000 sequencing, which was completed by Gene Denovo Biotechnology Co. (Guangzhou, China). All sequenced genes were blasted with the genome of *O*. *fragrans*.

### De novo assembly of RNA-Seq reads and quantification of gene expression

First, the adapter sequences were deleted from the raw reads. Then, low-quality reads (with over 40% of bases with quality scores of 10 or lower and/or over 10% of bases unknown) were deleted by using fastp version 0.18.0 (https://github.com/OpenGene/fastp) from each data set to establish more reliable results. Following this, clean, high-quality reads from all the samples were combined. Then an index of the reference genome was built, and paired-end clean reads were mapped to the reference genome using HISAT 2.2.4 (https://daehwankimlab.github.io/hisat2/) with ‘-rna-strandness RF’ and other parameters set as a default. The mapped reads of each sample were assembled by using String Tie version 1.3.1 (http://ccb.jhu.edu/software/stringtie/) in a reference-based approach^54,55^. For each gene, the expression level was measured by fragments per kilobase exon model per million mapped fragments (FPKM), based on the number of uniquely mapped reads, to eliminate the influence of different gene lengths and sequencing discrepancies in the gene expression calculation^3^.

### Identification and functional analysis of DEGs

Transcripts with a fold change > 2 and false discovery rate (FDR) < 0.05 were considered to be differentially expressed between growth stages. The gene sequences were compared with GO and KEGG databases^56–58^. The threshold was set to E-value < 1e−5, and the gene function information was obtained^59,60^. GO enrichment analysis and KEGG pathway enrichment analysis, the former being a standardized gene function classification system and the latter the main pathway analysis database, were performed for DEGs^61^. The annotations identified the signal transfusion pathways and related metabolic pathways of DEGs, which further revealed the biological functions of these genes^62^.

### Screening of hub genes and co-expression network regulation graphs

WGCNA was used to analyze the correlation between endogenous hormones and DEGs. The endogenous hormone content in *O*. *fragrans* was measured as a physiological indicator. A gene clustering tree was constructed according to the correlation between the expression levels of genes and the gene module. Modules were divided according to the clustering relationship between genes. The modules with similar expression patterns were combined according to the similarity of the module feature values. Genes with similar expression patterns were placed in the same module. We first determined the module most related to the character of interest. Based on KEGG annotation of the transcriptome, combined with the correlation analysis diagram of traits, corresponding feature modules were found. The MM (MM > 0.9) value of the characteristic module corresponding to key genes was set to further identify hub genes. To study the relationship between hub genes and edge genes, Cytoscape software was used to draw a regulatory network map. First, the downstream genes were sorted according to the weight value from the highest to the lowest. Then, according to the weight value (weight > 0.2), the TFs were selected to draw the regulatory network map.

### Verification of gene expression using qRT-PCR

To verify the accuracy of the transcriptome data, genes were selected from transcriptome data. Primers were designed using Primer 5.0 software (Table S3). *OfRAN* of *O*. *fragrans* was selected as the internal reference gene^63^. cDNAs were synthesized from 5 µg total RNA and diluted 20-fold for gene expression experiments. The qRT-PCR reaction kit used was SYBR Premix Ex Taq (Takara Biotechnology, Dalian, Liaoning Province, China). Reactions were performed at 95 ℃ for 3 min, followed by 40 cycles of 95 °C for 5 s, and 60 °C for 30 s. Relative gene expression levels were calculated according to the 2^−∆∆Ct^ comparative CT method^3^. Three technical replicates and three biological replicates were used for each sample.

## Conclusion

This is the first study of the transcriptome and physiology of *O*. *fragrans* callus*.* The content of endogenous hormones and transcriptome sequencing of *O*. *fragrans* calli were studied at different growth stages. The initial stages of callus growth were mainly regulated by JA, whereas the early stages of callus senescence were regulated by JA and IAA. The association between the co-expressed gene modules formed by DEGs and the endogenous hormones was analyzed by WGCNA. Here, we found that hub genes (*OfSRC2*, *OfPP2CD5*, *OfARR1*, and *OfPYL3*, *OfEIL3b*) involved in plant hormone signal transduction pathways could be regulated by four TFs (*OfERF4*, *OfMYC2a*, *OfTGA21c*, and *OfHSFA1*), which altered the JA and IAA content and regulated callus proliferation in *O*. *fragrans*. These results provide new insights for future research on the proliferation efficiency of *O*. *fragrans* callus.

## Supplementary Information

Supplementary Figure S1.

Supplementary Figure S2.

Supplementary Figure S3.

Supplementary Figure S4.

Supplementary Table S1.

Supplementary Table S2.

Supplementary Table S3.

## Data Availability

The transcriptome data have been uploaded to the NCBI Sequence Read Archive (https://www.ncbi.nlm.nih.gov/sra/) under accession number PRJNA760274. The datasets used and/or analyzed during the current study are available from the corresponding author on reasonable request.
