# Supplementary figures and images for: Integrated transcriptome and endogenous hormone analysis provides new insights into callus proliferation in Osmanthus fragrans
Source: Sci Rep. 2022 May 9;12:7609. doi: 10.1038/s41598-022-11801-9 (PMC9085794; doi:10.1038/s41598-022-11801-9)

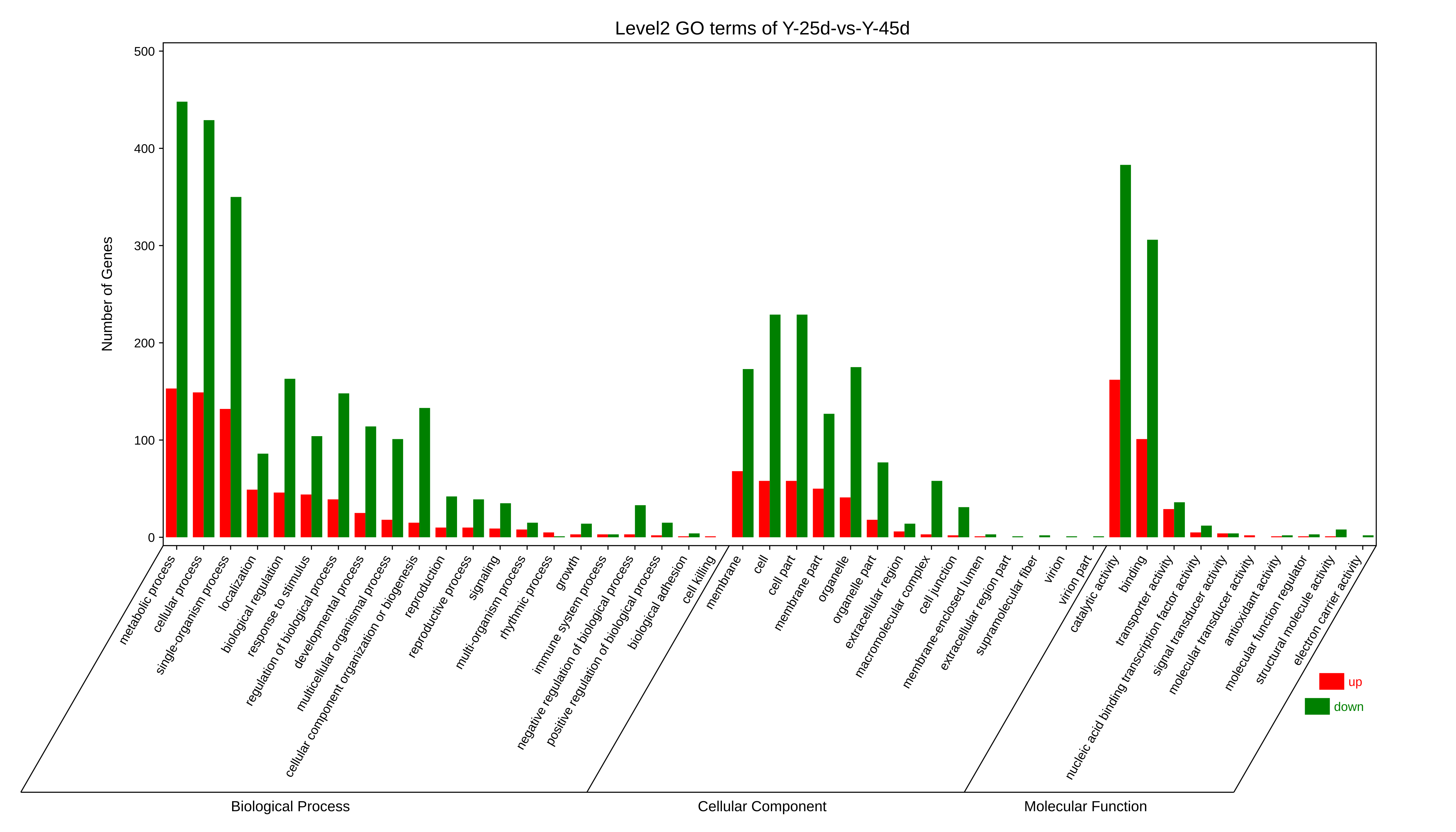

Supplement: Supplementary file 1 — Supplementary Figure S1. [file 41598_2022_11801_MOESM1_ESM.png]

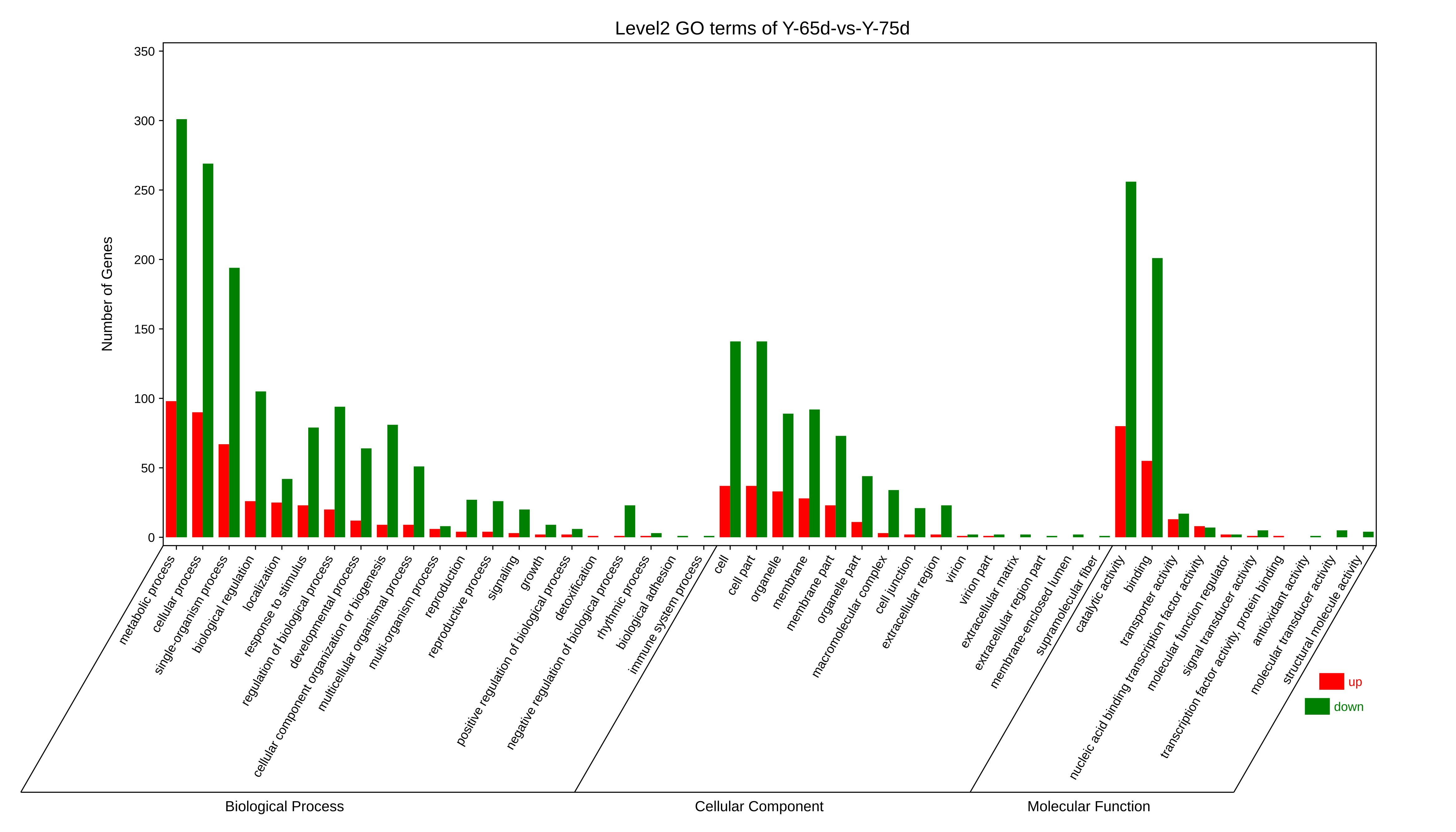

Supplement: Supplementary file 2 — Supplementary Figure S2. [file 41598_2022_11801_MOESM2_ESM.png]

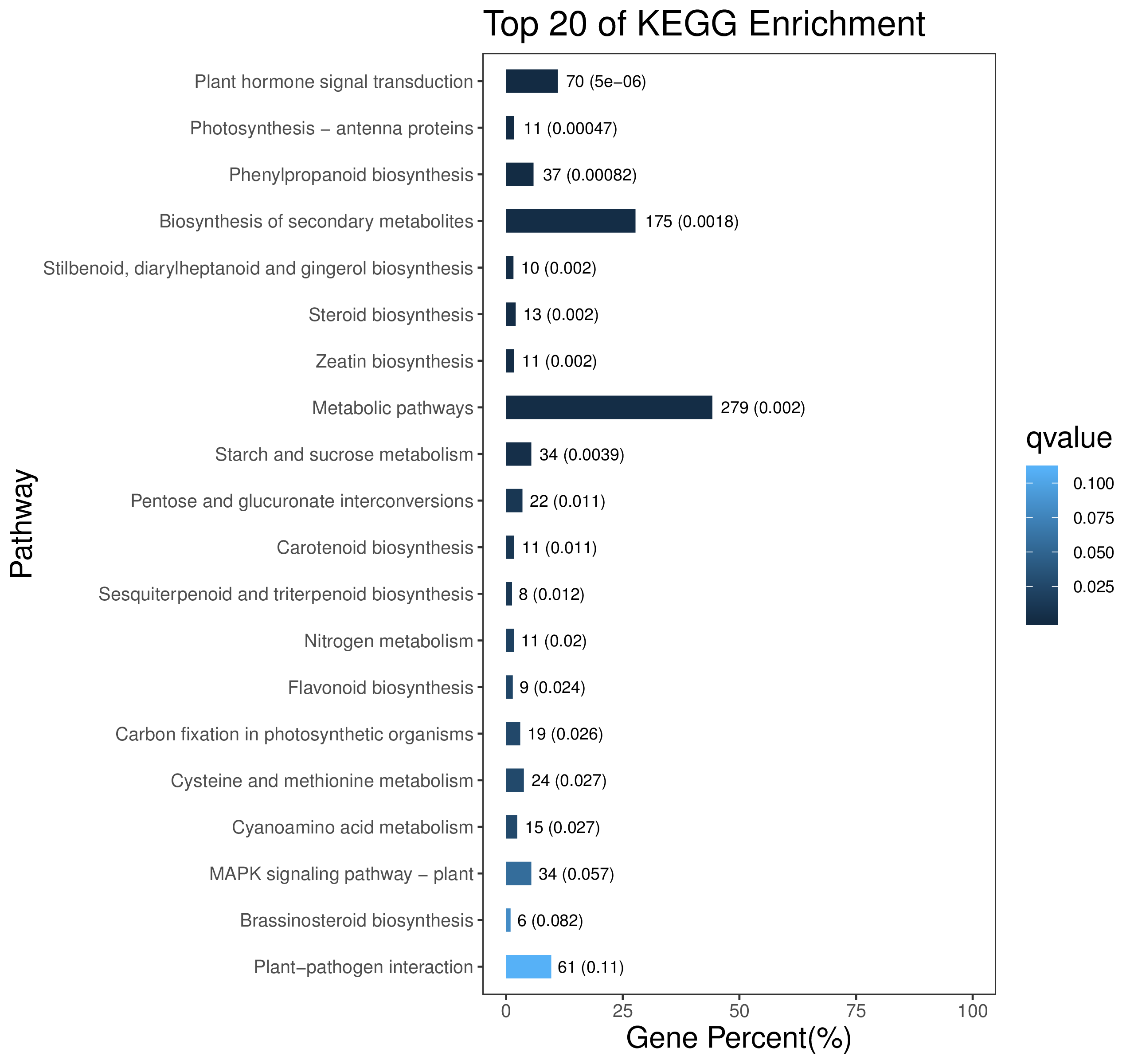

Supplement: Supplementary file 3 — Supplementary Figure S3. [file 41598_2022_11801_MOESM3_ESM.png]

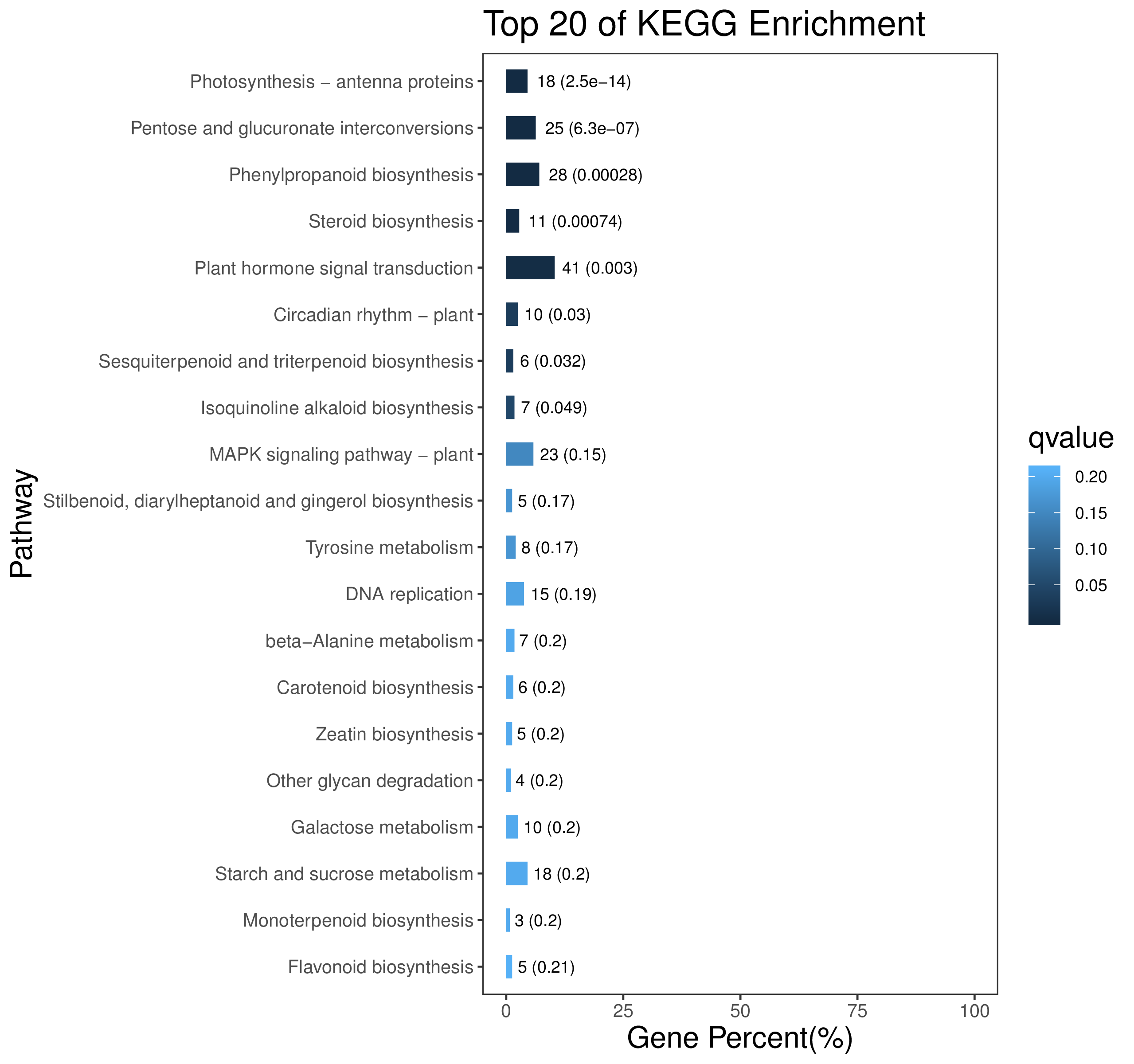

Supplement: Supplementary file 4 — Supplementary Figure S4. [file 41598_2022_11801_MOESM4_ESM.png]
