# Supplementary material for: Integrated transcriptome and endogenous hormone analysis provides new insights into callus proliferation in Osmanthus fragrans
Source: Sci Rep. 2022 May 9;12:7609. doi: 10.1038/s41598-022-11801-9 (PMC9085794; doi:10.1038/s41598-022-11801-9)
Supplement: Supplementary file 5 — Supplementary Table S1. [file 41598_2022_11801_MOESM5_ESM.docx]

**Table S1.** Principal component analysis of the five developmental stages of *O*. *fragrans* callus

| **Comparable group** | **Var ID** | **VIP** |
| --- | --- | --- |
|  | JA | 1.23371 |
|  | ABA | 1.22779 |
| Y-25d versus Y-45d | BR | 1.21934 |
|  | GA_3_ | 1.21067 |
|  | ZR | 0.13329 |
|  | IAA | 0.0138838 |
|  | JA | 1.10685 |
|  | ABA | 1.10635 |
| Y-45d versus Y-55d | ZR | 1.07881 |
|  | GA_3_ | 1.0077 |
|  | BR | 0.950858 |
|  | IAA | 0.68369 |
|  | ZR | 1.16576 |
|  | ABA | 1.15638 |
| Y-55d versus Y-65d | BR | 1.12482 |
|  | JA | 1.02063 |
|  | GA_3_ | 0.983738 |
|  | IAA | 0.170722 |
|  | JA | 1.0756 |
|  | IAA | 1.07485 |
| Y-65d versus Y-75d | ZR | 1.01676 |
|  | GA_3_ | 1.00356 |
|  | BR | 0.955842 |
|  | ABA | 0.856291 |

Y-25d: callus at 25 d; Y-45d: callus at 45 d; Y-55d: callus at 55 d; Y-65d: callus at 65 d; Y-75d: callus at 75 d. ABA: abscisic acid; BR: brassinosteroids; GA_3_: gibberellin; IAA: auxin; JA: jasmonic acid; ZR: zeatin.
