# Supplementary material for: Integrated transcriptome and endogenous hormone analysis provides new insights into callus proliferation in Osmanthus fragrans
Source: Sci Rep. 2022 May 9;12:7609. doi: 10.1038/s41598-022-11801-9 (PMC9085794; doi:10.1038/s41598-022-11801-9)
Supplement: Supplementary file 6 — Supplementary Table S2. [file 41598_2022_11801_MOESM6_ESM.docx]

**Table S2.** The gene ID of selected genes

| **Number** | **Gene ID** | **Name shown in this paper** |
| --- | --- | --- |
| 1 | evm.model.Contig182.90-gene | *OfTLP* |
| 2 | evm.model.Contig667.36-gene | *OfJHK* |
| 3 | evm.model.Contig31.60-gene | *OfLIP* |
| 4 | evm.model.Contig87.96-gene | *OfWOX11* |
| 5 | evm.model.Contig734.3-gene | *OfFXO* |
| 6 | evm.model.Contig55.196-gene | *OfDIR* |
| 7 | evm.model.Contig213.36-gene | *OfWOX8a* |
| 8 | evm.model.Contig311.64-gene | *OfPCA* |
| 9 | evm.model.Contig271.106-gene | *OfPSBO* |
| 10 | evm.model.Contig189.78-gene | *OfWOX13* |
| 11 | evm.model.Contig312.40-gene | *OfMADO* |
| 12 | evm.model.Contig667.30-gene | *OfTAR* |
| 13 | evm.model.Contig165.104-gene | *OfWOX8b* |
| 14 | evm.model.Contig177.34-gene | *OfCYP* |
| 15 | evm.model.Contig1.274-gene | *OfPP2CD5* |
| 16 | evm.model.Contig58.103-gene | *OfSRC2* |
| 17 | evm.model.Contig126.10-gene | *OfMYC2a* |
| 18 | evm.model.Contig126.11-gene | *OfMYC2b* |
| 19 | evm.model.Contig60.30-gene | *OfERF4* |
| 20 | evm.model.Contig383.14-gene | *OfERF5* |
| 21 | evm.model.Contig22.296-gene | *OfGTE7* |
| 22 | evm.model.Contig64.273-gene | *OfDREB1B* |
| 23 | evm.model.Contig126.54-gene | *OfHSFA4B* |
| 24 | evm.model.Contig159.2-gene | *OfEIL3a* |
| 25 | evm.model.Contig389.35-gene | *OfPYL3* |
| 26 | evm.model.Contig25.220-gene | *OfARR1* |
| 27 | evm.model.Contig421.18-gene | *OfEIL3b* |
| 28 | evm.model.Contig476.22-gene | *OfAHK3* |
| 29 | evm.model.Contig104.88-gene | *OfPP2CA* |
| 30 | evm.model.Contig663.11-gene | *OfWRKY4* |
| 31 | evm.model.Contig408.27-gene | *OfTGA21* |
| 32 | evm.model.Contig525.1-gene | *OfVIP1a* |
| 33 | evm.model.Contig408.27-gene | *OfTGA21c* |
| 34 | evm.model.Contig99.85-gene | *OfMYB78* |
| 35 | evm.model.Contig126.54-gene | *Of**DIVARICATAc* |
| 36 | evm.model.Contig97.116-gene | *OfGTE7* |
| 37 | evm.model.Contig63.113-gene | *OfNAC029* |
| 38 | evm.model.Contig268.78-gene | *OfLHW* |
| 39 | evm.model.Contig285.46-gene | *OfDIVARICATAb* |
| 40 | evm.model.Contig453.35-gene | *OfTAF12Ba* |
| 41 | evm.model.Contig63.64-gene | *OfMYB88* |
| 42 | evm.model.Contig541.2-gene | *OfTAF12B* |
| 43 | evm.model.Contig397.9-gene | *OfVIP1* |
| 44 | evm.model.Contig319.59-gene | *OfWRKY44* |
| 45 | evm.model.Contig92.177-gene | *OfHSFA1* |
| 46 | evm.model.Contig235.129-gene | *OfbHLH112* |
| 47 | evm.model.Contig50.17-gene | *OfAt3g21360* |
| 48 | evm.model.Contig208.84-gene | *OfNAC021a* |
| 49 | evm.model.Contig170.8-gene | *OfERF2* |
| 50 | evm.model.Contig175.76-gene | *OfCpNIFS3* |
| 51 | evm.model.Contig398.46-gene | *OfGTE4* |
| 52 | evm.model.Contig109.150-gene | *OfGT-2* |
| 53 | evm.model.Contig535.1-gene | *OfIDD2* |
| 54 | evm.model.Contig159.2-gene | *OfEIL3* |
| 55 | evm.model.Contig13.277-gene | *OfWRKY20* |
| 56 | evm.model.Contig288.46-gene | *OfDIVARICATAa* |
| 57 | evm.model.Contig136.152-gene | *OfbHLH143* |
| 58 | evm.model.Contig13.219-gene | *OfNAC021b* |
