# Supplementary material for: Integrated transcriptome and endogenous hormone analysis provides new insights into callus proliferation in Osmanthus fragrans
Source: Sci Rep. 2022 May 9;12:7609. doi: 10.1038/s41598-022-11801-9 (PMC9085794; doi:10.1038/s41598-022-11801-9)
Supplement: Supplementary file 7 — Supplementary Table S3. [file 41598_2022_11801_MOESM7_ESM.docx]

**Table S3.** Primer sequences used for qRT-PCR

| **Number** | - **Gene name** | **Primer sequence** |
| --- | --- | --- |
| 1 | *OfJHK* | F:GGCTCATCACCGTTCCACA |
|  |  | R:GGTAAATTCCGTCCAAGGCTAA |
| 2 | *OfLIP* | F:CATTTTGCTGTTCCCAGTCTCA |
|  |  | R:CGGCTTTAACGGGCTTGA |
| 3 | *OfPCA* | F:CAGCCCAGCAAGTAGGAGTTC |
|  |  | R:TGTATAAGCAGCTTCCATCATCACT |
| 4 | *OfTLP* | F:GCTTCCCTTTTCTTCCATTACAC |
|  |  | R:CCCATTGCAGTCTCCTGTTTG |
| 5 | *OfTAR* | F:GGCTCATCACCGTTCCACA |
|  |  | R:AAGGCTAAATACGGGCGAGA |
| 6 | *OfFXO* | F:GACGAATCGGAGCGACAAA |
|  |  | R:GGCGGGTAGAATCCTTTGC |
| 7 | *OfCYP* | F:TGTTGATCGTCGGGCTTTC |
|  |  | R:CCACTTGCCTGATCGGGTT |
| 8 | *OfMADO* | F:TGTTGTTGGGCTTGGCATT |
|  |  | R:CTCCTCGCACAGCTCCATTT |
| 9 | *OfPSBO* | F:GCTACTCTGATGCAGCCAACC |
|  |  | R:GCAAGAGCAAACCCAGCAAT |
| 10 | *OfWOX8a* | F:GAATAAGCAGCCGCCACAA |
|  |  | R:CATCACTTTAACAAACATCCCTCC |
| 11 | *OfWOX8b* | F:TAACGGAGTAGTGAATAATGGAGGA |
|  |  | R:ACAAATGGCGGAGTAAACAGC |
| 12 | *OfWOX13* | F:GAGCGAGGTTTGGAAATCTGTAT |
|  |  | R:TGAAGTTGGACTGGTGTTGGAG |
| 13 | *OfWOX11* | F:AGTCTTAGAGTTAGAGGCGGTGAA |
|  |  | R:AGCTTGAGGACAGAGGGAAATT |
| 14 | *OfPIF* | F:GCTAGTTGGAGATGTCCCTTGG |
|  |  | R:GCTCGTGTTTGAACCCTTTGA |
| 15 | *OfSRC2* | F:TAAGGGCAACATCAAGTTTTCG |
|  |  | R:GCTGGATACGCAGTCATAGGC |
| 16 | *OfERF4* | F:TGTTGGAGGTCACGTTCTTGTT |
|  |  | R:TGCTGTCCATTTGGCATCAC |
| 17 | *OfPP2CD5* | F:CGGAATGATGGGTTGTTATGG |
|  |  | R:TGCCGATGGCGAAGTGTAG |
| 18 | *OfMYC2a* | F:GGAGTAACGAGGAGGGCATG |
|  |  | R:TACTCGTCTTCCCAGCACCC |
| 19 | *OfPP2CA* | F:CCGAAGGAGGAGGATGGAA |
|  |  | R:GACGATGGCGATGCAGATAA |
| 20 | *OfEIL3a* | F:CGTGCCTTCCTCACCTCTTG |
|  |  | R:AGTTGGAGTGACCACCTGCTTT |
| 21 | *OfPYL3* | F:AGTAGACACCACAAGCACCAGC |
|  |  | R:CCCTCACCAATAGCCAAACAA |
| 22 | *OfEIL3b* | F:AAAAGGTGTTTAAGGTGGATAGCA |
|  |  | R:TCCAACTCTTCCGGTTCAATCT |
| 23 | *OfAHK3* | F:GGGGCGGATATGGATAAAATT |
|  |  | R:CACCCAAACCGATGCTATGA |
| 24 | *OfARR1* | F:TTCACGGGCTTACAAGGGAG |
|  |  | R:TAGCGAGGCTTTGTGGTGG |
| 25 | *OfHSFA1* | F:GAGGGTTGAGTGTTGGGAGC |
|  |  | R:ACGATGCTGATGGTATGCTCC |
| 26 | *OfTGA21c* | F:AGACTTGCCCAAAATCGTGAA |
|  |  | R:TTGCCACTGCCAGAATGAGA |
| 27 | *OfRAN* | F:AGAACCGACAGGTGAAGGCAA |
|  |  | R:TGGCAAGGTACAGAAAGGGCT |
